# “Health without Borders”: Early Findings and Lessons Learned from a Health Promotion Program for Ethnic Minorities Living in Italy

**DOI:** 10.3390/ijerph20095646

**Published:** 2023-04-26

**Authors:** Serena Barello, Marta Acampora, Lorenzo Grimaldi, Cecilia Maccacaro, Sara Dell’Acqua, Barbara Spina, Daniela Giangreco

**Affiliations:** 1 EngageMinds HUB—Consumer, Food & Health Engagement Research Center, Department of Psychology, Università Cattolica del Sacro Cuore, Milano and Cremona, L.Go Gemelli 1, 20123 Milan, Italy; marta.acampora@unicatt.it (M.A.); sara.dellacqua@gmail.com (S.D.); 2 Italian League Against Cancer, Via Giacomo Venezian 1, 20133 Milan, Italy; lorenzo.grimaldi@legatumori.mi.it (L.G.); cecilia.maccacaro@legatumori.mi.it (C.M.); barbara.spina@legatumori.mi.it (B.S.); daniela.giangreco@legatumori.mi.it (D.G.)

**Keywords:** health promotion, patient engagement, transcultural medicine, transcultural health, community-based health education, participatory health, oncology, cancer prevention, ethnic minorities, multicultural health promotion

## Abstract

In multicultural contexts, health promotion can be challenging due to people’s differences in beliefs, values, and practices regarding health and healthcare. Using the prototypical case scenario offered by the “Health without Borders” program, this study was generally aimed at summarizing the lessons learned and suggesting implications that are hopefully relevant to future culturally competent health promotion programs. This exploratory study used in-depth interviews, focus groups, and document analyses as primary methodological tools to gather data. A qualitative approach was chosen because it has the potential to explore, in depth, the main characteristics (values, operational domains, and action strategies) behind this prototypical case. The study findings suggest that the multicultural health promotion program under study is characterized by four main intertwined core values (i.e., empowerment; peer education; social embeddedness; tailor-made). In turn, these values are expressed in the ten main operational domains (i.e., proactive approach to health promotion; fostering interculturality in health promotion; fostering multidisciplinarity in health promotion; measuring the impact of initiatives; identifying, training, and activating key community members in the role of peer educators; promoting community engagement; fostering a “domino effect”; building institutional links with the organization of the territory; continuous training of the professionals involved in the initiatives; flexibility and a constant focus on projects’ continuous redesign) that orient specific strategies of action. This program is based on a tailor-made principle for intervention design and delivery. This feature allows intervention providers to flexibly incorporate the target population’s values in delivering health promotion activities. Therefore, the value of this prototypical case lies in the design of “adjustable” initiatives that fit the “program-as-designed” with the cultural characteristics of target populations involved in the intervention.

## 1. Introduction

The 21st century has been characterized by the process of globalization and the emergence of multicultural societies. This phenomenon requires a deep revision of the established healthcare approaches. Although no single definition of culture is universally accepted by social scientists, there is general agreement that culture is learned, shared, and transmitted from one generation to the next. Culture can be observed in a group’s values, norms, practices, systems of meaning, ways of life, and other social rules. In such a group, these and other factors may be directly or indirectly associated with health-related behaviors and/or with acceptance and adoption of health promotion programs and messages.

Health promotion in a multicultural setting involves promoting the health and well-being of individuals belonging to ethnic minorities with culturally and linguistically diverse backgrounds [1,2]. This can include addressing cultural beliefs and practices that may impact health behaviors and outcomes [3,4], as well as promoting the cultural competence of healthcare providers [5].

Several studies have examined the effectiveness of health promotion interventions with ethnic minorities [6,7]. Among others, a systematic review [8] found that culturally-tailored health promotion interventions were generally more effective at improving health behaviors and outcomes compared to non-tailored interventions. Another systematic review [9] found that health promotion interventions incorporating cultural beliefs and practices were more effective at improving the health behaviors and outcomes of women from ethnic minority groups. 

Cultural competency is also an important factor in the success of health promotion interventions with cultural minorities [10]. A study on this topic [11] found that a cultural competency training for healthcare providers was associated with improved user satisfaction and trust, as well as better health outcomes. In addition, one’s own culture may influence the conceptualization of health and disease through other culturally related variables, such as migration status, demographic, socioeconomic, and educational background [12,13,14]. 

Specifically, there is a need for more studies that explore the mechanisms by which cultural tailoring and cultural competency may contribute to improved health behaviors and outcomes, above all, in health promotion programs for cancer prevention [14,15].

To effectively supply culturally competent health promotion programs, providers must be able to identify and understand the various cultures and subcultures within a given population, as well as how these cultures relate to healthcare and preventive behaviors. 

They also need to be able to adapt their actions and strategies to new trends and representations of health in an intercultural context. This requires adaptation to new forms of health communication and to new plural and multicultural contexts while taking a culturally appropriate and person-centered approach [16,17]. Providing health promotion interventions can be challenging in any healthcare environment, but it is especially difficult in a multicultural setting, as there may be fewer commonalities in terms of norms and values related to health. To provide person-centered care to someone from a culture different from one’s own, healthcare practitioners must be more aware of their own preconceptions and prejudices, and avoid the “cultural expertise” model, which risks stereotyping a person based on only one aspect of their cultural identity. Instead, they should take a culturally competent approach that emphasizes recognizing individuals’ dynamic complexity through the exploration of one’s own multifaceted, context-dependent cultural identity [18].

*The “Health without Borders” Program: A Prototypical Case Scenario for health promotion for cancer prevention with ethnic minorities*

The “Health without Borders” program took its first steps in 2017. The project was born from a pilot health education activity dedicated to foreign women. Thanks to this first activity, it was possible to detect a growing interest in foreign communities on the topic of cancer prevention, which led to the second and third edition of “Health without Borders”. This led to involving the entire population including the male population, notoriously more reticent in addressing issues related to cancer prevention. More in detail, the “Health without Borders” program has been realized between 2018 and 2022 in northern Italy by LILT APS (i.e., the Italian League against Cancer—Milano, Monza, and Brianza—Social Promotion Association (https://www.legatumori.mi.it/, accessed on 20 January 2023). LILT APS is devoted to sustaining the promotion of healthy lifestyles through methodologies that are attentive to the cultural, linguistic, and social characteristics of ethnic minorities living in Italy. The program, currently in its third edition, has involved participants from Egypt, Morocco, Tunisia, Algeria, Senegal, Cameroon, the Philippines, Colombia, Peru, and Ecuador and has now reached a total of 1642 persons. The project is the result of a collaboration between LILT health specialists, the Ismu Foundation (https://www.ismu.org/en/, accessed on 20 January 2023), and EngageMinds HUB Research Center (www.engagemindshub.com, accessed on 20 January 2023). Moreover, it actively involved the Islamic Center of the city of Monza, the Orthodox Church of Pavia, the Diocese of Migrants, and the consulates of Colombia and Ecuador. 

Ethnic minorities living in Italy amount to about 5 million people (https://www.statista.com/statistics/798256/number-of-foreign-residents-in-italy/, accessed on 20 January 2023). According to the Word Cancer Report 2020, migrants living in Italy are diagnosed with cancer 20% more than Italians, and only one out of two resident immigrants adheres to screening programs.

Despite the obvious need to improve health promotion activities devoted to cancer prevention, targeted to ethnic minorities living in Italy, it is often difficult for the third sector and public organizations to effectively engage in this target when taking care of one’s own health is often not the individual’s top priority. It is therefore essential to develop health promotion initiatives by adopting appropriate and engaging methodologies capable of being culturally competent and having effective social impacts. LILT carries out health education programs based on the assumption that primary prevention is the best weapon to fight cancer and prevent various non-communicable diseases. The adoption of measures capable of achieving health goals is considered a priority and can be achieved by moving from mere cultural mediation to the development of community empowerment and people’s involvement in health-related issues [19,20]. The general aim of the “Health without Borders” program is to promote healthy behaviors among ethnic minorities living in Italy and motivate individuals to become the main players in the management of their well-being. More specifically, the actions implemented by this program are described here, as follows:

(1) Training peer educators (namely “LILT Health Ambassadors”) on health issues within the communities to which they belong, so that they can engage their peers in bottom-up health promotion initiatives.
(2) Conducting seminars on health-related topics involving the foreign communities involved in the program, based on the specific information needs reported by the LILT Health Ambassadors.
(3) Developing and providing capacity building trainings for health workers in contact with ethnic minorities.
(4) Developing and providing a toolbox of training materials translated into the languages of the ethnic minorities involved in the program to promote culturally competent health promotion.
(5) Engaging and training LILT Health Ambassadors as key community members. LILT Health Ambassadors work with the program managers and are responsible for identifying communities’ health needs, raising awareness of cancer prevention, and promoting LILT initiatives; moreover, they orient community members to LILT services, and give feedback to LILT managers for continuous co-planning of health promotion interventions. Health ambassadors are selected for their prominent role in the reference community. In the project, most of the ambassadors are women—but there are also some men—who belong to associations or religious centers, and are leaders and charismatic people. In fact, they often hold top positions within their communities.

Using the prototypical case of a health promotion intervention offered by the “Health without Borders” program, we conducted a study with the general aim of summarizing the lessons learned from this experience in terms of its theoretical and pragmatic implications. Therefore, the aim of this study is twofold:

(1) To outline the conceptual background and strategies featuring the “Health without Borders” program for a more systematic and sustainable approach to culturally competent health promotion interventions targeted to ethnic minorities.
(2) To share lessons learned from this program and to suggest implications that are relevant to other health promotion programs willing to take into account cultural factors related to health and healthcare.

## 2. Materials and Methods

### 2.1. Study Design

This exploratory study used in-depth interviews, focus groups, and written records as primary methodological tools for data collection. A qualitative approach was chosen for analyzing this prototypical case of health promotion intervention because it has the potential to explore, in depth, its main characteristics, and identify meanings and processes that are relevant to its implementation with ethnic minorities.

For this study we used the method of focused ethnography, which is mainly used to study a specific topic or experience. In fact, it differs from traditional ethnographic research in that the object of study is circumscribed and can be identified a priori. This method can be used to study institutions and often focuses on a group of participants with similar characteristics. In this case, what is relevant are the common behaviors and experiences of stakeholders who have experienced the “Health Without Borders” program from different perspectives. 

### 2.2. Sample

To improve the validity of a study conducted according to an ethnographic approach, members from various groups of a study population should be involved as key informants. We included key informants willing to provide rich information regarding their experiences in the development of the health promotion project, and other general informants who could provide less intense content [20].

Informants were obtained using purposive sampling and included a selection of key members of the project committee employed by LILT, LILT Health Ambassadors, some participants in the proposed initiatives, and healthcare professionals in charge of delivering seminars on health-related topics. In addition, professionals from other institutions interested in implementing the LILT Ethnic Minority Health Promotion Program were interviewed to discuss the conditions for the feasibility and transferability of the program.

### 2.3. Data Collection

**Figure 1:**
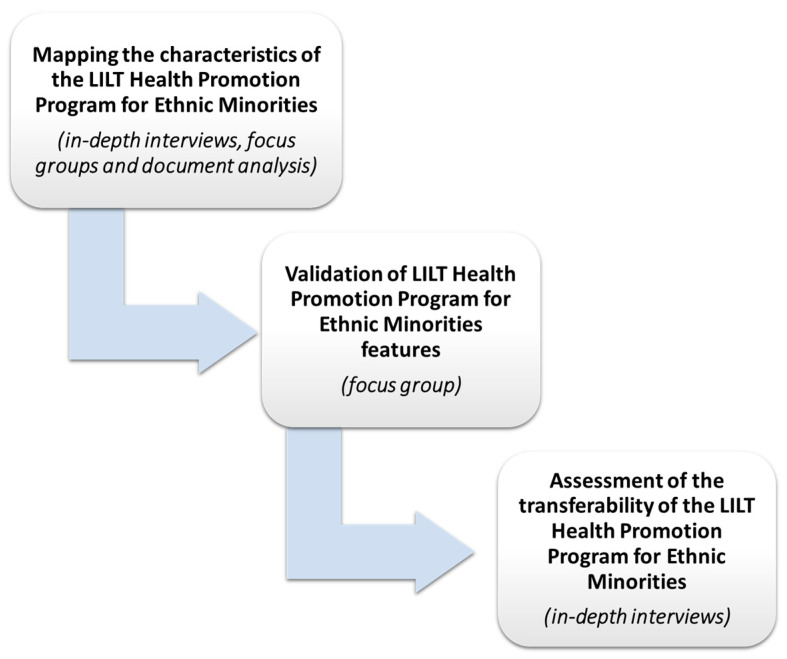
Methodological steps for data collection.

A detailed description of the study’s methodological steps follows (see Figure 1).

A. *Mapping the characteristics of the LILT Health Promotion Program for Ethnic Minorities.* In order to map and collate the characteristics of the program, this first phase involved conducting in-depth interviews, focus groups, and document analysis. In particular, the following were involved:
(a) A total of two online, three-hour long focus groups were conducted by two researchers trained in qualitative methodologies (S.B. and M.A.) and involved a total of nineteen participants each, balanced according to the role they played in the program (i.e., five LILT project managers, four health professionals, five Health Ambassadors from the Filipino, Arabic-speaking, Sub-Saharan, Romanian, and Latin American communities and five simple participant representatives of the ethnic minorities involved in the project). These focus groups followed a flexible guideline of conduction aimed at reconstructing the pinnacles of the intervention program from the perspectives of the involved stakeholders. The focus groups were conducted in Italian as the participants were all fluent in this language.
(b) Then, a total of 13 online, in-depth individual interviews were conducted following a flexible guideline of conduction with other Health Ambassadors from the Filipino, Arabic-speaking, Sub-Saharan, Romanian, and Latin American communities (n= 10) and health professionals—1 gynecologist, 1 dietitian, and 1 oncologist (n = 3)—involved in delivering the health-related seminars during the program. The interviews were conducted in Italian as the participants were all fluent in this language.
(c) Finally, a systematic document analysis of all the descriptive material produced by LILT about the program was carried out with the aim of integrating what emerged from the discussion groups and in-depth interviews.
B. *Validation of LILT Health Promotion Program for Ethnic Minorities features.* A second phase of the research consisted of conducting an additional online focus group with a total of 8 participants, with a balanced number of LILT project managers (n = 4) and health ambassadors (n = 4), with the aim of presenting the characteristics and pillars of the program according to the findings of the previous phases and to reach a consensus on its essential characteristics (epistemological, theoretical, and methodological).
C. *Assessment of the transferability of the LILT Health Promotion Program for Ethnic Minorities*. Finally, we conducted a total of 9 online, in-depth, qualitative interviews, each lasting approximately one hour, with stakeholders from health and social care organizations (having similar characteristics to LILT and with an interest in replicating the working method), with the aim of testing the transferability and feasibility of the program.

### 2.4. Data Analysis

**Figure 2:**
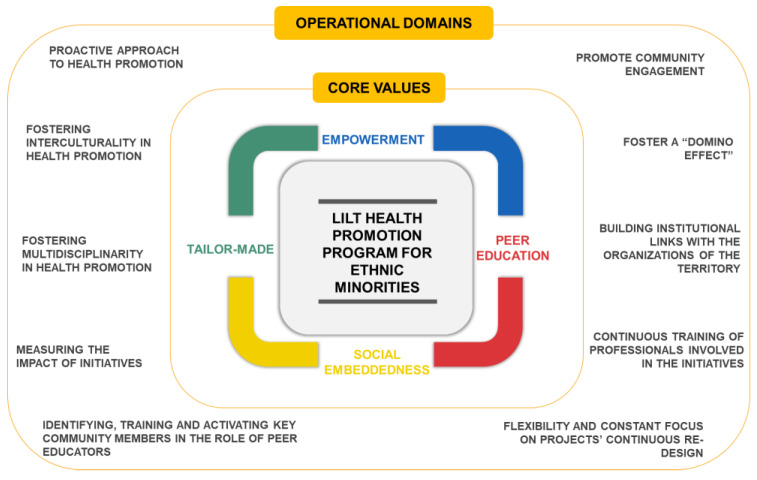
Core values and operational domains featuring the LILT Health Promotion Program for Ethnic Minorities.

Using an ethnographic approach [21], we analyzed interviews, focus groups, and documents; the analysis entailed line-by-line coding conducted by 2 researchers (S.D. and M.A.) who held regular discussions with a senior researcher (S.B.) to resolve any discrepancies in the coding. This thematic analysis allowed us to describe the constitutive pillars of the culturally sensitive health promotion program (namely, LILT Health Promotion Program for Ethnic Minorities) (Figure 2), and we selected quotes from the interviews and documents that further described each element of the program. For domain analysis, the data were read several times, looking for references to issues related to the characteristics of health promotion interventions. Several possible headings emerged, such as types of organizational culture and administrative support. Next, domain analysis worksheets were created to help visualize the structure of each domain, including the umbrella terms and semantic relationships. In the taxonomic analysis, a domain with large data was selected and a search was made for similarities between all the terms included under the selected domain, based on the same semantic relationship. To ensure the trustworthiness and confirmability of results, we constantly involved participants, preventing the conceptual categories emerging from the analysis. The conceptual model describing the values and operational domains featuring the “Health without Borders” program has been discussed in a formal meeting with all the involved stakeholders to check its validity.

## 3. Results

According to the results obtained, the LILT Health Promotion Program for Ethnic Minorities is characterized by four main intertwined core values. These values, in turn, are expressed in ten main operational areas that guide specific action strategies. Figure 2 summarizes the characteristics of the program. 

In the next paragraphs, a detailed description of core values, operational domains, and strategies of action is reported. 

### 3.1. Core Values

The analysis of the collected material resulted in the identification of four main core values that guide the LILT Health Promotion Program for Ethnic Minorities.

**Empowerment**: The intervention should strengthen the internal resources of both the community and individual members. Any health promotion initiative and communication is carried out within the community, and in the typical gathering places of the foreign community to which the initiative is addressed (e.g., Islamic centers, Orthodox churches, migrant associations, consulates).

*“We need to make the community feel part of the change. Supporting self-efficacy and involving people directly in managing their health is always the best strategy for promoting health”. (ID002, LILT project manager, female, 54 years old)*

**Peer education**: The intervention should promote the activation of key community members (i.e., ‘health ambassadors’), who have demonstrated leadership within the community, communication skills, and have expressed confidence, to actively participate in the project and promote and educate others on health-conscious behaviors.

*“When your peer talks to you about sensitive issues such as health, you certainly feel freer to ask questions and more confident in what you are told”. (ID003, Health Ambassador, Sub-Saharan community, male, 45 years old)*

**Social embeddedness**: The intervention should involve people with similar characteristics (age, gender, ethnicity). It eases networking to create strong channels for information transfer and community involvement in health promotion initiatives. One Health Ambassador said the following:

*“When the initiatives are tailor-made for the socio-cultural target you are talking to, everything works better”. (ID007, Health Ambassador, Filipino community, male, 37 years old)*

**Tailor-made**: The intervention should be adapted to the cultural characteristics of the target group. Each group has its own cultural values, traditions, beliefs, and practices that influence their health beliefs and behaviors.

*“Health can be viewed differently by people, even according to their own culture, and this needs to be taken into account when changing a habit/belief about these issues.” (ID001, healthcare professional, gynecologist, female, 47 years old)*

*“When thinking about the structure of meetings, you have to bear in mind that people have deeply rooted beliefs and habits, often linked to their culture. It could be counterproductive to try to impose your ideas without taking these aspects into account. The best thing is to look for a point between two poles, creating a complementarity between the different ideas”. (ID004, LILT project manager, male, 29 years old)*

### 3.2. Operational Domains and Specific Strategies of Action

The results of the interviews and focus groups suggested ten main operational domains with specific action strategies that characterize the nature of the LILT program. These domains are described here, as follows.

**Promote community engagement**. The first domain that characterizes the program is the promotion of community engagement. Indeed, the intervention envisages a strong involvement of the participants who become the subjects of the health promotion initiative. This objective can be pursued by involving and activating cultural mediators (i.e., Health Ambassadors) who can act as interlocutors with the community on health-related issues, taking into account the socio-cultural dimensions that influence the behavior of the population concerned.

*“It’s important to touch the emotional side of the participants and deal with relevant issues to get their attention”. (ID013, health professional, nutritionist, female, 32 years old)*

*“Health ambassadors are key to engaging people who would be very difficult to reach in any other way”. (ID016, LILT project manager, female, 27 years old)*

**Proactive approach to health promotion.** Another key area of the intervention program is to take a proactive approach to promoting the health of the target community. This means not waiting for people to seek help at health facilities but trying to reach them directly in their communities. This would promote the penetration of outreach initiatives and their spill-over into people’s daily lives, thereby increasing the likelihood of healthy behavior change. A particularly relevant strategy for achieving this goal is to promote collaboration and a network approach with places of worship, migrant associations, consulates, and voluntary organizations in the area where health promotion initiatives are to be implemented.

*“You have to go to people’s homes to engage them, to physically bring them to the meetings”. (ID007, Health Ambassador, Filipino community, male, 37 years old)*

*“The meetings are held in places where we usually go, and that definitely helps people to participate”. (ID019, participant, Arabic-speaking community, female, 56 years old)*

**Generate a “domino effect”.** A third characteristic area of the program is the desire to generate a “domino effect” in the health promotion processes. Indeed, the program foresees a continuous exchange of materials, which will allow the replication of awareness-raising and training initiatives also in the immediate network of those who have participated in them. This aspect is facilitated by the use of a website and social media to increase the visibility of the content and initiatives proposed to the community.

*“I shared what I learned and the materials they gave us with my friends and relatives”. (ID018, participant, Latin American community, female, 54 years old)*

*“It might be useful to open up some meetings to anyone who wants to attend”. (ID002, LILT project manager)*

**Fostering interculturality in health promotion**. A fourth key domain of the intervention model is the constant orientation towards the promotion of interculturality in health promotion initiatives. The LILT program is based on an open relationship with people from different cultures, seeking to adapt initiatives to their specific needs and to enhance the contribution of each individual with his or her particularities, without imposing a single point of view on the topics to be trained. This can be enabled by promoting moments of encounter that encourage the inclusion of people from different cultures. The aim is to foster the achievement of the common good and to promote intercultural dialogue on health promotion.

*“You should not put yourself in a position of authority, but you should welcome what comes from the people”. (ID011, Health Ambassador, Romanian community, female, 35 years old)*

*“It’s essential to get to know each other’s point of view by letting people speak”. (ID018, participant, Latin American community, female, 54 years old)*

**Fostering multidisciplinarity in health promotion**. Encouraging a multidisciplinary approach to health promotion is the fifth key dimension of the program. In fact, all the initiatives carried out under the “Health without Borders” program require the collaboration of experts from different health disciplines to provide the population with information that covers several areas of interest. Of relevance is the involvement of health professionals who have a cultural and ethnic background similar to that of the community they are targeting. In addition, the use of cultural mediators can be another effective strategy.

*“The involvement of professionals from different backgrounds is a great enrichment for the project”. (ID023, health professional, oncologist, male, 47 years old)*

*“The key figures in the project include different professionals such as psychologists, nutritionists, gynecologists, surgeons, but also community members”. (ID002, LILT project manager, female, 54 years old)*

**Identifying, training, and activating key community members**. Training health ambassadors in the role of peer educators is an added area of the model. In fact, in the experience of the stakeholders interviewed, these individuals are a key bridge in easing the transmission and absorption of information about health and health-promoting behaviors in foreign communities. The ability of the organizations promoting these initiatives to carefully select the individuals to whom they entrust this role, and to provide them with proper training and skills are of paramount importance.

*“Health ambassadors are absolutely crucial, but they need to be properly trained to spread LILT’s mission”. (ID004, LILT project manager, male, 29 years old)*

**Building institutional links with the organization of the territory**. It is also essential to establish institutional links and networks with the organizations in the area where such initiatives are carried out. The fundamental element of this area is the creation of relationships of trust between the main actors of the territory. This will generate partnerships with third parties to create opportunities for visibility and the implementation of further health promotion initiatives. This pillar would promote the wide dissemination of initiatives in the community of interest, thus increasing their effectiveness.

*“It is essential to develop a relationship of trust with LILT and its practitioners. This is the key to success”. (ID011, Health Ambassador, Romanian community, female, 35 years old)*

*“It is necessary to create a real network that will allow us to reach out to the people…without waiting for the people to come to us”. (ID004, LILT project manager, male, 29 years old)*

**Flexibility and constant focus on projects’ continuous redesign**. Project reflexivity and flexibility are further key dimensions of the LILT approach. The intervention’s activities are indeed designed to be constantly adapted to the field’s needs from time to time. This is achieved by adapting content to participants’ evolving information needs, listening to community members, and being flexible and willing to change direction when needed.

*“Health promotion for minority communities is an activity that grows over time and is constantly changing to meet the needs that are continuously changing”. (ID018, participant, Latin American community, female, 54 years old)*

**Continuous training of professionals involved in the initiatives**. The LILT approach requires professionals involved in the design and implementation of initiatives to be trained in both content and communication style. This must be accompanied by a sincere passion and commitment to their professional activities. Training processes that support them in their role as health promoters must therefore be provided on an ongoing basis. Training these actors on content skills, but also on communication approaches capable of involving people from different cultural worlds and backgrounds, is particularly important here too.

*“It is important that the professionals have a sense of ownership of the project”. (ID023, healthcare professional, oncologist, male, 47 years old)*

*“Practitioners need to be trained, not only in content, but also in how to present it”. (ID001, healthcare professional, gynecologist, female, 47 years old)*

**Measuring the impact of initiatives**. Finally, the LILT approach recognizes the crucial importance of measuring the impact of initiatives so that they can be continually optimized. In fact, the “Health Without Borders” project provides different moments for analyzing the impact of the interventions on the participants. This is essential for monitoring the objectives achieved and those that still need to be worked on. These include measuring the impact on knowledge during and at the end of the sessions, monitoring access to prevention services, and promoting the follow-up of initiatives to test the results in the long run and sustain behavioral change in the long term.

*“Regarding the measurement of effects, it is important both to get feedback from participants but also to develop a systematic post-intervention measurement”. (ID004, LILT project manager, female, 54 years old)*

### 3.3. Barriers to the Program’s Implementation

A number of issues emerged as possible barriers to the implementation of the LILT program according to stakeholders from other institutions interested in implementing the program.

There was a lack of experience in health promotion project development. Some organizations stated that they had no experience in health, prevention, and related issues. They suggested that in order to overcome this barrier, they should be supported through training initiatives that highlight the specifics of health promotion projects and explain how this issue is relevant when working with ethnic minorities. They also recognized a lack of information on how health has different connotations for different cultures.

*“We are not experienced in dealing with health. We have never had any training on these issues or how they are relevant to foreign communities. There should be training for us; these are important issues”. (ID026, project manager, non-profit association, female, 32 years old)*

**Lacking experience in dealing with multiculturalism**. Another possible barrier to implementing this intervention could be that personnel are not experienced in dealing with multicultural issues, and therefore lack awareness about how to adapt content and how to raise awareness and provide training.

*“We’ve never had experience with foreign communities, we simply lack the skills”. (ID025, project manager, non-profit association, male, 56 years old)*

**The lack of links with cultural mediators and health professionals from foreign communities**. The lack of health professionals from the foreign community can also be an obstacle to health promotion interventions. This is because the cultural competence of those responsible for promoting good health practices is crucial.

*“We have never done multicultural projects that allowed us to make contacts, especially with foreign people, so we don’t know any cultural mediators or health workers”. (ID028, project manager, non-profit association, female, 51 years old)*

## 4. Discussion

The purpose of this study was to analyze a prototype case of a health promotion intervention with ethnic minority populations living in Italy, to identify its distinctive elements, and to share the findings with the scientific and professional community interested in the subject. It was possible to identify the basic values and operational fields of action by analyzing the intervention model underlying the program studied. Making health promotion science responsive to the needs of specific populations, such as ethnic minorities, requires extending existing scientific models to include cultural variables and issues of cultural adaptation in health promotion interventions [22]. For instance, “Health without Borders” adapted the contents of the informational sessions to the specific cultural target, proposing different diet-related recommendations, and taking into consideration the cultural specificities related to food habits. Such cultural variables include language, belief systems and values, and conservative traditional community norms. These extended models and their empirical evaluation can inform the development of science-based interventions for such populations. LILT has developed a hybrid health promotion intervention program. This bridges the need for fidelity with the need for cultural relevance and specificity. In this approach, based on a tailor-made principle for intervention design and delivery, the core components of the intervention serve as the basis for the adapted program, while the program also incorporates the values of the target population to help refine the core components and develop new ones. The strength of this program, therefore, lies in the design of ‘adaptable’ initiatives that adapt the model program to the culture of the target ethnic minority, thanks to the active participation of the target population in the design of the initiatives [23]. Effectively implementing health promotion interventions into multicultural communities is an increasing priority for health researchers. The successful dissemination and implementation of effective interventions ensures that major health funders can demonstrate a return on investment in biomedical and behavioral research, and that all populations benefit from scientific discoveries to the greatest extent possible. However, evidence-based programs are rarely planned with dissemination and implementation in mind, leaving these interventions fundamentally mismatched with real-world contexts. Furthermore, while some evidence-based health promotion programs have been effectively modified for use in multicultural populations, only a few examples of such adaptations have been reported. There is a scarcity of literature on the acceptance and implementation of evidence-based health promotion interventions in multicultural communities, and the feasibility of scaling up effective programs is poorly understood, thereby exacerbating health inequities. The “Health without Borders” partners are generating data on community-responsive and engaged interventions that are also designed to facilitate dissemination and implementation efforts, reducing the time between research and practice to benefit multicultural communities, should these interventions prove effective. In this manuscript, we have provided an overview and the key barriers to the dissemination and implementation of science when working with multicultural communities. We then used the “Health without Borders” case study to highlight strategies that other organizations may use to plan for the scale-up and implementation of such a health promotion program. As a novel approach and a prototypical example of a health promotion intervention, it has several limitations, such as the limited generalizability of the results and the difficulty of bringing together heterogeneous health cultures. Moreover, the case is related to a specific Italian region (i.e., Lombardy), which is very different from other health and care realities in Italy and abroad regarding health services in terms of service accessibility or gratuity of health interventions. Therefore, comparisons with other realities may be difficult. For this reason, we tested the possible transferability of the program to other realities. In addition, a different mix of ethnicities among participants may have had an impact on the obtained results. The influence of the research process on the implementation process is also a limitation. Such intensive fieldwork always influences what happens in the field.

## 5. Conclusions

Health promotion practitioners are constantly faced with the dilemma of how to develop effective health promotion strategies to improve the health of their communities. It has been argued that the relevance of an individual’s community and cultural background is one of the many factors that influences health promotion outcomes. Overall, the literature suggests that promoting health among cultural minorities is more effective when it is culturally tailored and when providers are culturally competent. Further research is needed to understand the specific factors contributing to the effectiveness of these interventions, and to identify the best practices for promoting health among diverse cultural groups. Several reviews [3,24,25,26,27,28,29] have reported the effects of interventions aimed at improving health in migrant populations, with clinically relevant outcomes. However, little is known about the operational model behind these interventions. The results of our study are expected to improve the understanding of current health promotion studies targeting ethnic minorities. In addition, this study is particularly relevant as these populations will be considered in the context of achieving target 10 of the UN 2030 Agenda for Sustainable Development to reduce inequalities. Future research is needed to figure out how program activities affect health outcomes over time. In addition, as technology has been shown to be an important tool in overcoming pragmatic barriers to people’s engagement in similar initiatives, future developments of the program should consider incorporating technology to support engagement in health promotion interventions [30]. 

## Data Availability

Data are available upon reasonable request by contacting the corresponding author.
